# Histone modifications in embryo implantation and placentation: insights from mouse models

**DOI:** 10.3389/fendo.2023.1229862

**Published:** 2023-08-04

**Authors:** Shilei Bi, Zhaowei Tu, Dunjin Chen, Shuang Zhang

**Affiliations:** ^1^ Key Laboratory for Major Obstetric Diseases of Guangdong, Department of Obstetrics and Gynecology, The Third Affiliated Hospital of Guangzhou Medical University, Guangzhou, China; ^2^ Guangdong-Hong Kong-Macao Greater Bay Area Higher Education Joint Laboratory of Maternal-Fetal Medicine, Guangzhou, China; ^3^ Guangdong Engineering and Technology Research Center of Maternal-Fetal Medicine, Guangzhou, China

**Keywords:** histone modifications, epigenetics, embryo implantation, placentation, mouse models

## Abstract

Embryo implantation and placentation play pivotal roles in pregnancy by facilitating crucial maternal-fetal interactions. These dynamic processes involve significant alterations in gene expression profiles within the endometrium and trophoblast lineages. Epigenetics regulatory mechanisms, such as DNA methylation, histone modification, chromatin remodeling, and microRNA expression, act as regulatory switches to modulate gene activity, and have been implicated in establishing a successful pregnancy. Exploring the alterations in these epigenetic modifications can provide valuable insights for the development of therapeutic strategies targeting complications related to pregnancy. However, our current understanding of these mechanisms during key gestational stages remains incomplete. This review focuses on recent advancements in the study of histone modifications during embryo implantation and placentation, while also highlighting future research directions in this field.

## Introduction

The journey of new life begins with the formation of an embryo, which must be implanted in the uterus to establish a functional interaction between mother and fetus in mammals (1). Embryo implantation and placentation are key steps in the establishment of this communication and are required for a successful pregnancy. As the blastocyst acquires implantation competency, the endometrium differentiates to become receptive to the embryos under the regulation of estrogen (E2) and progesterone (P4) (2). Upon completion of implantation, the outer trophectoderm (TE) of the blastocyst begins to differentiate and forms the placenta, a transient organ that acts as a barrier between the mother and fetus (3). The placenta facilitates the exchange of nutrients and oxygen while protecting the fetus from harmful substances (4). Disturbance to implantation and placentation may lead to pregnancy-related complications, such as recurrent pregnancy loss, infertility, pre-eclampsia, fetal growth restriction, preterm birth, and stillbirth (5). Thus, a better understanding of the underlying molecular networks of embryo implantation and placentation will help to advance our understanding of the causes of pregnancy complications.

Epigenetic control is the process by which gene expression is regulated by chemical modifications to DNA and its associated proteins, without altering the underlying genetic code (6). These modifications act like switches, turning genes on or off, and allowing cells to respond to changing environmental signals, and have been implicated in a wide range of biological processes, including development, aging, and disease (7). It includes a range of chemical changes, such as DNA methylation, non-coding RNA (ncRNA) expression, and histone modification we aimed at in this review. Histones can be modified in a variety of ways, including methylation, acetylation, phosphorylation, ubiquitylation and poly (ADP)-ribosylation (8). These histone modifications affect chromatin compaction and accessibility of transcription factors or cofactors, thereby regulating gene transcriptional activation or silencing. The enzymes involved in catalyzing this histone modification can be classified as "writer" and "eraser". "Writer" refers to the enzymes that add histone modifications to histones, such as histone methyltransferases, histone acetyltransferases, and so on. “Eraser” removes specific modifications, including histone demethylases and histone deacetylases (9). The study of histone modification has been a rapidly growing field in recent years, and have discovered that changes in histone modification patterns are associated with a range of diseases, including cancer, cardiovascular disease, and neurodevelopmental disorders (10, 11).

**Figure 1:**
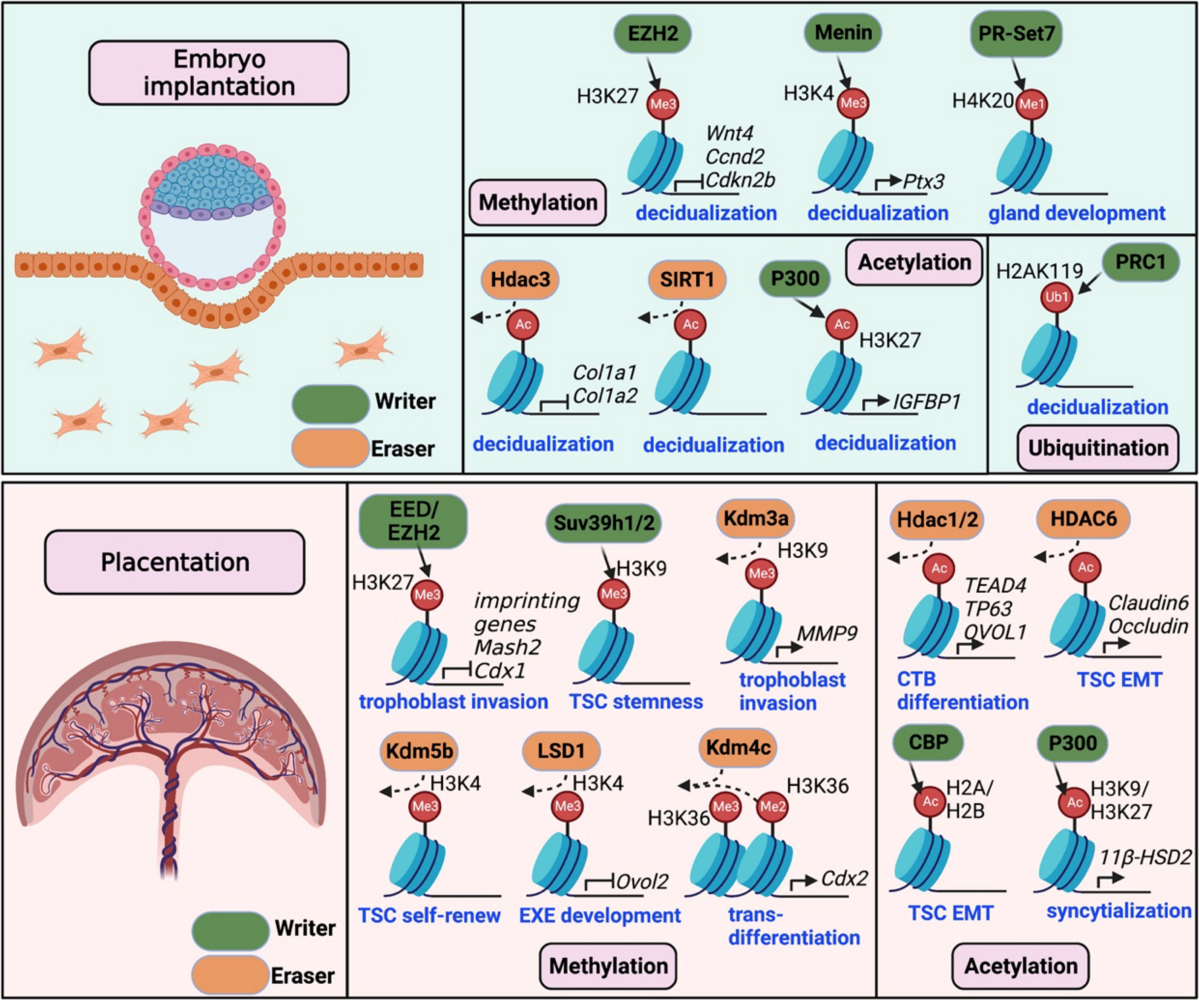
Histone modifications required for mouse embryo implantation and placentation. The upper panel illustrates the crucial writers and erasers involved in histone methylation (EZH2, Menin, and PR-Set7), histone acetylation (Hdac3, Sirt1 and P300), and histone ubiquitination (PRC1) during mouse embryo implantation. These modifications contribute to stroma decidualization by regulating critical gene expression (e.g., *Wnt4*, *Ccnd2*, *Cola1/2*, *Ptx3*, and *IGFBP1*). In the lower panel, histone modifications, including methylation and acetylation, are shown to be essential for placentation. Changes in histone methylation by writers (EED/EZH2, Suv39h1/2) or erasers (Kdm3a/4c/5b, LSD1) control gene expression required for trophoblast stemness, self-renew, invasion and differentiation. Acetylation regulators, including histone acetylases (CBP/P300) and deacetylases (Hdac1/2/6), are involved in TSC epithelial-to-mesenchymal transition (EMT) and differentiation.

Recent studies have revealed that histone modifications play critical roles in determining the success of embryo implantation and placenta development, providing valuable insights into the underlying molecular mechanisms. In this review, we present the current findings of implantation and placentation events in various model systems and in humans, primarily focusing on the impact of histone modifications on the embryo-uterus dialogues during gestation (**Figure 1**). A better understanding of the roles of histone modification in these processes may reveal new predictive and therapeutic targets for pregnancy-related complications.

## Histone modifications in implantation

The process of embryo implantation in both humans and mice involves a series of pivotal events, including 1) the establishment of receptivity in the uterine cavity; 2) the attachment reaction between the trophoblast and endometrial epithelium; 3) the interplay between trophoblast cells, endometrial epithelium, and stromal cells during the invasion of trophoblast cells and initiation of decidualization (12). Despite variations in the manner of blastocyst invasion into the endometrial lining among different species, many features of the implantation and decidualization process exhibit a commonality. Consequently, the molecular regulation of implantation and decidualization holds many similarities between humans and model organisms (1). Due to experimental difficulties and ethical restrictions, our understanding of human implantation still relies predominantly on animal models, particularly the mouse. The Cre-loxP system represents a potent investigative tool for discerning the physiological functions of genes across various organs (13). To conduct effective targeted knockout studies, it is necessary to utilize tissue-specific promoters to drive the expression of Cre recombinase (**Table 1**). Utilizing genetically modified mouse models and human clinical tissues, epigenetic regulations have been proven important in regulating the establishment of endometrial receptivity, blastocyst attachment and subsequent decidualization in recent years.

**Table 1:** Mouse models expressing cre recombinase in the uterus.

Cre model	Expression	References
*Pgr*	Epithelium, stroma, gland, myometrium	(14)
*Ltf*	Epithelium	(15)
*Sprr2f*	Epithelium	(16)
*Wnt7a*	Epithelium	(17)
*Amhr2*	Stroma, myometrium	(18)
*Smmhc*	Myometrium	(19)
*Sm22*	Myometrium	(20)

### Histone methylation

H3K27me3 exerts a repressive effect on gene transcription (21). To investigate the importance of H3K27me3 in decidualization, researchers isolated primary endometrial stromal cells (ESCs) from human endometrial tissues and induced decidualization in *vitro*. Transcriptome and epigenome profiles of the cells were obtained, revealing certain alterations in H3K27ac and H3K27me3 at the promoter regions of genes critical for decidualization, such as WNT4, ZBTB16, PROK1, and GREB1. This finding suggests the significance of H3K27me3 in the decidualization process (22). Brosens’s group has reported a significant decrease in the level of H3K27 methylation at the promoter distal end of decidual markers PRL and IGFBP-1, during the process of endometrial stroma-decidua transformation in human endometrium (23). Furthermore, studies have established that the generation and erasure of H3K27me3 in decidual stromal cells during pregnancy plays a critical role in the maintenance of gestation (24). The transcriptional silencing of specific gene targets by H3K27me3 helps to maintain uterine quiescence and prevents decidual expression of parturition-inducing hormone receptors, manifesting type 1 immunity and generating myofibroblasts for wound-healing responses. On the other hand, genome-wide H3K27 demethylation in late gestation allows for target gene upregulation and decidual activation, leading to labor entry (24).

Polycomb repressive complex 2 subunit (Ezh2), a core component of polycomb repressive complexes 2, is a histone methyltransferase that catalyzes mono-, di-, and tri-methylates histone H3 at lysine 27 (25). Given the extensive function of H3K27me3 in the process of implantation, researchers use *Pgr-cre* mouse model to conditional delete *Ezh2* in the uterus in order to further explore the roles of H3K27me3 in implantation (26–29). Osokine et al. provide evidence of the critical role of Ezh2 in the regulation of wound healing responses in the decidual stromal cells during early pregnancy. They demonstrated that Ezh2 confers resistance to TGF-β-mediated wound healing signals in the decidual cells. Conversely, the loss of EZH2 from the decidua leads to fibroblast activation, the induction of TGF-β target genes, and ultimately, pregnancy failure (28). H3K27me3-related gene silencing is canceled after EZH2 deletion, leading to dysregulated cell-cycle regulators expression, causing severe epithelial and stromal differentiation defects and failed embryo invasion (29). Unlike those studies using *Pgr-Cre* to conditional knockout *Ezh2*, deletion of *Ezh2* using *Amhr2-Cre* leads to marked defects in uterine adenogenesis. The deficient uterine adenogenesis was accompanied by impaired uterine function and pregnancy loss (30). These findings confirm the essential roles of *Ezh2*-mediated H3K27me3 in implantation.

The H3K4me3 modification can be methylated by the mixed-lineage leukemia MLL1/2 complex and is predominantly located in the gene transcription start region, where it functions as a transcriptional activator (31). Menin, as a crucial component of the MLL complex, can regulate gene transcription through H3K4me3 (32). Liu et al. utilized a combination of genetic, biochemical, and pharmacological approaches to prove that *Men1* conditional knockout in uterus disrupts the appropriated differentiation of decidual stromal cells through regulating the expression of *Ptx3* in an H3K4me3 dependent manner. The reduction in *Ptx3* expression subsequent to *Men1* ablation leads to the aberrant activation of ERK1/2, resulting in a decrease in BMP2 induction and impaired decidualization (33). *Pr-set7*, as a methyltransferase of H4K20me1, has been reported to have a crucial role in regulating gland development of uterus and promoting the maintenance of pregnancy (34).

### Histone acetylation

As early as the 1970s, scholars had confirmed that treatment with E2 in the mouse and rat uterus resulted in an increase in histone acetylation levels (35, 36). Histone deacetylase blockers and HDAC inhibitors enhance proliferative and morphogenetic effects of E2 on the mouse uterus (37, 38). Moreover, H3K27ac is upregulated during decidualization and is significantly enriched at the distal promoter of the decidualization marker IGFBP1 (39), These findings suggest that histone acetylation plays a crucial role in regulating uterine receptivity and decidualization during implantation, possibly through modulation of ER and PR in response to E2 and P4.

Hdac3 is a class I histone deacetylases (Hdac) family, which is dependent on Zn^2+^ and responsible for transferring the acetyl group from acetylated histone proteins (40). Conditional ablation of *Hdac3* using *Pgr-cre* mouse model resulted in infertility caused by impaired implantation and decidualization. The loss of *HDAC3* caused decidualization defects due to abnormal activation of *Col1a1* and *Col1a2* genes, and this regulatory mechanism is conserved in human endometrium (41). In addition, the application of HDAC inhibitors to endometrial stromal cells enhances the histone acetylation levels of the TIMP-1 and TIMP-3 promoter regions, promoting gene transcription and suppressing the invasion of trophoblast (42).

Sirtuin1 (SIRT1) is a member of the sirtuin family of Class III histone deacetylases which has been reported to be involved in the regulation of cellular senescence and aging (43). Sirt1 mediates the deacetylation of histones and non-histone proteins in an NAD^+^-dependent manner (44). Regarding histones, SIRT1 exhibits the ability to deacetylate several lysine residues, including lysine 26 of histone H1 (H1K26), lysine 9, 14, 18, and 56 of histone H3 (H3K9, H3K14, H3K18, and H3K56), and lysine 6, 12, and 16 of histone H4 (H4K6, H4K12, and H4K16). By modulating the acetylation status of these specific lysine sites, SIRT1 plays a crucial role in regulating the transcription of relevant genes (45, 46). In addition to its impact on histones, SIRT1 also targets numerous non-histone proteins, including various transcription factors and co-factors. Notable examples include p53, HSF1, HIF-1α, NF-κB, p300, and KAT5. Through deacetylation, SIRT1 directly influences the functions of these proteins, thereby exerting broader effects on cellular processes (47–51). Recent studies using the *Pgr-cre* mouse model have confirmed that deletion of *Sirt1* resulted in impaired decidualization and pregnancy failure (52, 53). Moreover, the uterus exhibits signs of premature aging after *Sirt1* deletion (52). Interestingly, utilizing *Pgr-cre* to achieve uterine *Sirt1* overexpression also caused decidualization abnormalities and pregnancy failure (54). SIRT1 overexpression in endometriotic lesions worsens endometriosis development, but the application of Sirt1 inhibitor EX527 shows improvement (54). Although EX527 has already undergone a clinical trial for Huntington's disease and its safety was validated (55), the safety of EX527 for a developing embryo or fetus is a major concern. Therefore, further verification is needed to determine whether EX527 can be a promising drug for patients with pregnancy failure caused by decidualization abnormalities.

The process of human decidualization is associated with a significant increase in histone acetylation levels in the promoter regions of decidualization markers, such as IGFBP-1 and PRL. This increase makes transcriptional factors more accessible, thus promoting transcription (56, 57). Further research revealed that during decidualization, C/EBP and FOXO1 bind to the enhancer region of IGFBP-1, promoting its expression and increasing H3K27ac levels with the help of C/EBP's recruitment of p300 (39). Application of histone acetylation inhibitors in the uterine endometrial cells promoted the expression of glycodelin, a secretory protein that is induced by progesterone exposure during the early secretory phase following ovulation and remains present until the start of menstruation or the establishment of pregnancy (38, 58). HOXA10 is a crucial transcription factor that regulates uterine receptivity during implantation (59). HOXA10 can be acetylated by PCAF at K338 and K339 sites, which subsequently suppresses the expression of ITGB3 in endometrial epithelium, hindering embryonic implantation (60).

### Histone ubiquitination

The Polycomb Repressive Complex 1 (PRC1) is an essential chromatin-based repressor of gene transcription which catalyzes monoubiquitination of histone H2A (H2AK119ub1) and stabilizes H3K27me3 modification. The PRC1’s catalytic center is comprised of the RING1A or RING1B protein and one of the six PCGF proteins (61). Inhibition of H2AK119ub1 disrupted the normal progression of decidualization, confirming the critical role of H2AK119ub1 mediated by PRC1 in decidualization (62). Xin et al. also reported that BMI1, a crucial component of PRC1, interacts with PR and the E3 ubiquitin ligase E6AP in a manner independent of PRC1, and mediates PR ubiquitination, allowing the uterus to respond to P4 during the establishment of uterine receptivity. Additionally, aberrantly low expression of BMI1 was observed in endometrial samples of patients with recurrent implantation failure, providing a potential target for the treatment of recurrent-implantation-failure (63).

### Histone lactylation

Lactylation modification is a novel protein post-translational modification type induced by lactate that was first reported in 2019, and studies have shown that lactylation modification plays an important role in cancer, immunity, and other fields (64–66). Yang et al. found that during the pregnancy of sheep, the lactate content in the endometrial tissue and embryo significantly increased, as well as the H3K18la modification. The target genes regulated by H3K18la are mainly related to GSH metabolism and function. Moreover, the application of inhibitors to block glycolysis leads to a decrease in implantation capability in mice, while the application of lactate significantly rescued implantation capability. These findings suggest that histone lactylation plays an important role in the implantation process (67).

## Histone modifications in placentation

As the blastomeres proceed with development, the initial determination of cell fate commences, culminating in the gradual formation of the inside cells and outside cells, ultimately giving rise to two distinct lineages, the ICM and TE (68). During this period, cells from both lineages undergo loss of totipotency and are constrained to their respective lineage differentiation trajectories (69). In this fate-determining process, covalent histone modifications play critical roles. *In vitro* and in *vivo* experiments have demonstrated that deletion of SETDB1, a H3K9 methyltransferase, in mouse embryonic stem cells (mESCs) induces their differentiation towards the TE lineage (70, 71). SUV39H1, another H3K9 methyltransferase, also suppresses ICM-associated gene expression during TE lineage development (72, 73). These studies indicated that the commitment of ICM versus TE is precisely regulated by the incorporation of H3K9me3 at specific chromatin domains by distinct histone methyltransferases. In addition to H3K9me3, H3K27me3, as a transcriptional repressive-associated histone modification, also plays a critical role in TE and ICM lineage differentiation (72, 73). There is a significant difference in the global H3K27me3 level between ICM and TE. In the ICM lineage, promoter regions of TE-associated genes are enriched with H3K27me3 modification, while H3K27me3 enrichment on *Cdx2* and *Gata3* genes gradually decreases during TE lineage differentiation and development (72, 73). These findings suggest the importance of H3K27me3 in cell fate determination. Interestingly, *Hdac1* binds to self-renewal-related genes *Oct4*, *Sox2*, and *Nanog* in the ICM lineage, and to lineage-regulating genes *Cdx2*, *Elf5*, and *Eomes* in the TE lineage, accompanied by transcriptional activation-associated modification H3 acetylation (74). These findings suggest that although the two lineages have differences, they also share similarities, such as Hdac1 binding to target genes in different lineages to maintain their respective self-renewal abilities (74).

The development of the placenta starts with contact between the blastocyst TE and the endometrial epithelium in both humans and mice. The blastocyst penetrates the endometrium *via* its trophoblastic cells and embeds deeply, accompanied by an abundance of proliferation and differentiation of these cells, which constitutes the primary placenta structure (3). Although both mouse and human placentas share a hemochorial nature, they exhibit distinct variations in their overall morphology (75).

In humans, upon contact with the decidual, a portion of trophoblastic cells undergo fusion to form primary syncytium, which penetrates the decidual layer and embeds deeply (4, 76). The trophoblasts with stemness undergo differentiation to form functional trophoblast subtypes, including cytotrophoblasts (CTB), syncytiotrophoblasts (STB), and extravillous trophoblasts (EVT) to fulfill the placental functions (77). These cells gradually form a villous-like structure, composed of fetal vessels and mesenchymal stroma. The CTB and STB which are located at the outer layer of the placental villi, absorb nutrients from the maternal blood and form the maternal-fetal barrier that provides protection for the fetus (77, 78). Meanwhile, the EVT migrates and invades the decidual layer and even the myometrium to anchor the placenta firmly in the uterus and remodel the spiral arteries of the uterus to establish maternal-fetal blood circulation (76–78).

In mice, TE continues to proliferate to form extraembryonic ectoderm (EXE) and the ectoplacental cone (EPC). The extraembryonic mesoderm appears at E6.5 and gives rise to the allantois and the extraembryonic mesodermal layers of the amnion and chorion (3, 79). At E8.5, the fusion of the allantois with the chorion constitutes a key event in placenta maturation. The fusion facilitates the invagination of blood vessels derived from the extraembryonic mesoderm into the trophoblastic layer of the chorion, forming the key structure of the placenta-the labyrinth layer (79). As the labyrinth layer of the placenta expands, the trophoblasts differentiate into syncytiotrophoblasts (STB) and sinusoidal trophoblast giant cells (TGC) to fulfill their respective roles in placental function (3).

Although mice and humans have different placental structures and key developmental genes, conditional knockout mice remain the main model for investigating placental development due to their shared classification as hemochorial placentas and the convenience of genetic manipulation (4). The mouse models that express Cre specifically in different trophoblast cell types are used to investigate the function and potential mechanisms of different genes (**Table 2**).

**Table 2:** Mouse models expressing cre recombinase in the trophoblast.

Cre model	Expression	References
*Elf5*	All subtype	(80)
*Tpbpa*	Tpbpa-lineage	(81)
*Gcm1*	STB	(82)
*Cyp19*	All subtype	(83)
*Tpbpar/Adaf-AdaP*	All subtype	(84)
*Plf*	TGC	(85)
*Pl1*	TGC	(85)
*Tat*	All subtype	(86)

### Histone methylation

During embryonic development, cells undergo the first lineage specification event, leading to the formation of either the embryonic or extra-embryonic lineage, the latter of which will develop into the placenta. In this process, there is a widespread loss of H3K27me3 in promoter regions, followed by rapid dynamics during the morula to blastocyst transition. (87, 88). Yang et al. reported that H3K27me3 and DNA methylation regulates key developmental genes in embryonic and extra-embryonic cells. This helps to maintain the highly regulated developmental plasticity in the embryonic cells, as well as restricting the developmental potential of the extra-embryonic cells (89). During pluripotent cell development, many genes bound by PRC2 have both the repressive H3K27me3 mark and the activating H3K4me3 mark. The H3K4me3/H3K27me3 bivalent state is absent in developmental genes until lineage differentiation initiates, and most bivalent genes are repressed (90). This regulatory mechanism allows genes to be silenced as key developmental regulators while being primed for future activation (or repression), and thus generally transition to monovalent configurations upon differentiation. In the embryonic lineage, PRC1 and poised RNAP are not recruited to the PRC2-bound genes. However, in the extra-embryonic lineage, bivalent genes are selectively targeted by Suv39h1-mediated H3K9me3 repression, which determines the initial fate (72).

Maternal H3K27me3-mediated imprinting can mediate gene silencing in DNA hypomethylated regions during embryonic development (88). In trophoblast, the paternal imprinting of the Kcnq1 and IC2 domain on the distal end of mouse chromosome 7 is mainly achieved through H3K27me3 and H3K9me2 mediated by Polycomb complex, rather than relying on DNA methylation (91–93). Moreover, oocyte-derived H3K27me3 also plays a vital role in maintaining non-canonical gene imprinting in extraembryonic lineage cells (94). The significance of Polycomb complex in gene imprinting has been demonstrated through the whole-body knockout mouse model and triploid compensation experiments, which revealed that Eed knockout resulted in abnormal expression of the paternal imprinting gene *Mash2* (95). Animal cloning can be achieved through somatic cell nuclear transfer (SCNT), but the success rate is relatively low and often results in abnormal placental development. H3K27me3-mediated gene imprinting may be a key factor (96). Additionally, aberrant upregulation of H3K27me3-mediated clustered miRNAs from Sfmbt2 has been identified as the major cause of abnormal placental hyperplasia in SCNT mice (97).

During the process of placenta maturation until the end of pregnancy, there are substantial changes in the epigenome of cytotrophoblasts. Lv et al. have reported that in trophoblast, EZH2 suppresses CDX1 expression to regulate its invasion through an H3K27me3-dependent manner (98). To further investigate the impact of EZH2 on placental development, Nugent et al. used *Cyp19-cre* to conditionally knockout *Ezh2 in the* placenta. After EZH2 deletion, female fetuses became more vulnerable to prenatal stress. But the placental morphology and function changes after EZH2 knockout remain obscure (99).

H3K9 methylation is a histone modification associated with transcriptional repression and is reported to regulate the function of trophoblast (100, 101). Kdm3a is a demethylase that specifically targets H3K9 methylation. Chakraborty et al. have demonstrated that under hypoxic conditions, HIF downregulates H3K9 methylation at the Mmp12 promoter through Kdm3a, which promotes transcriptional expression of MMP9 and enhances trophoblast invasion (100). Meanwhile, methyltransferase Suv39h2 regulates H3K9 methylation and is involved in the pluripotency and differentiation of mTSC. Knockout of SUV39H2 in mTSC leads to significant changes in the landscape of H3K9 methylation and triggers differentiation (101).

H3K4me3 is predominantly enriched at the transcription start site of active genes to activate transcription (102). KDM5B, as a demethylase of H3K4me3, regulates the self-renewal and H3K4 methylome in mTSC (103). Lsd1 can erase out monomethyl and dimethyl groups from H3K4me2/3 or H3K9me2/3 to activate or silence gene transcription (104, 105). Knockout of Lsd1 leads to abnormal development of EXE and ultimately embryo death (106). It has been demonstrated that *Lsd1* directly suppresses the expression of Ovol2 to maintain proper development of the EXE (106). *Lsd1* also regulates mitochondrial homeostasis in mTSC through the target gene *Sirt4* (107). Furthermore, *Lsd1* forms a complex with *Jdjm2b* and *Tfap2c* at critical gene promoters to activate transcription and safeguard the identity of mTSC (108). The abnormal H3K4me3 in the extraembryonic lineage caused by assisted reproductive technology (ART) procedures also results in developmental abnormalities. Knocking down the modifying enzyme *Kmt2e* can restore the expression of the corresponding genes and promote embryonic development (109). Besides, H3K4me3 expression is also found downregulated in human preeclampsia placenta (110).

A recent study reported that human iPSCs can be induced to differentiate into trophoblast-like stem cells (111), which has important implications for both basic research and potential clinical applications. Interestingly, Yu et al. found that H3K36me2 modification plays a critical role in regulating the induction of pig iPSCs into trophoblast-like stem cells. *Kdm4c*, as a demethylase of H3K36me3/2, activates the expression of *Cdx2* to promote this trans-differentiation process (112).

### Histone acetylation

The establishment of a model using human trophoblast stem cells (hTSC) is of great importance for investigating the processes of trophoblast proliferation and differentiation during human placental development (113). In this context, the addition of the histone deacetylase inhibitor VPA proved to be particularly effective in promoting hTSC proliferation during the establishment phase, while other inhibitors like SAHA or TSA showed similar effects. These findings underscore the significance of histone acetylation in maintaining the pluripotency and proliferation of hTSCs, although the precise underlying mechanism remains unclear. During the differentiation of primary cytotrophoblasts, significant decreases in H3K27ac, H3K14ac, and H3ac are detected. This observation has been further validated in the Bewo, an epithelial cell line isolated from the placenta of a patient with choriocarcinoma (114). Moreover, the examination of placental tissues from pregnancy-related diseases revealed alterations in H3K8ac, H3K27ac, H3K9ac, and the histone deacetylase SIRT1 to varying extents, indicating a potential involvement of histone acetylation in the pathological mechanisms underlying placental diseases (110, 115–118).

Using *Sirt1*-null embryos and established mTSC, it was discovered that mTSC were unable to differentiate properly following *Sirt1* knockout (119). Similarly, Xiong et al. achieved trophoblast-specific knockout of *Sirt1* using *Elf5-cre* and observed the same phenotype, indicating that *Sirt1* knockout might activate key genes in trophoblasts and disrupt their developmental trajectory (120). In addition, Histone deacetylase 1 and 2 have been found critical in driving hTSC differentiation by controlling the expression of *TEAD4, TP63, OVOL1* and *CGB* (114). Additionally, HDAC6 has been proven required for trophoblast stem cell differentiation *via* directly deacetylates histones on the epithelial gene promoters such as Claudin 6 and Occludin during epithelial-to-mesenchymal transition (EMT) of TS cells (121).

Besides deacetylases, histone acetyltransferases have also been found important in trophoblast. Cbp is widely expressed and capable of acetylating both histone and non-histone proteins (122). Abell and colleagues discovered that the absence of Map3k4 in mTSC leads to EMT, through a mechanism in which the knockout of Map3k4 prevents the direct phosphorylation of Cbp by JNK, thereby promoting the acetylation of histone proteins H2A and H2B and facilitating the transcription of relevant genes (123). P300 is an originally identified coactivator of CBP. It plays a crucial role in acetylation of H3K9 and K3K27 associated with 11β-HSD2 expression in syncytiotrophoblasts (124).

### Other histone modification

Citrullination is a post-translational modification (PTM) that is catalyzed by the peptidyl arginine deiminase (PAD) enzyme family, which includes PADI1-4 and PADI6 (125). Recent findings shed light on the significance of histone citrullination. Ballasy et al reported that *Padi2* and *Padi3*, the most widely expressed members of the PAD enzyme family in mTSCs, play a crucial role in regulating differentiation. Knockout of *Padi2* and *Padi3* resulted in decreased expression of *CDX2* and *SOX2*, leading to a bias towards trophoblast giant cell (TGC) differentiation. Additionally, deletion of *Padi2* and *Padi3* had a substantial impact on the epigenomic landscape of mTSC, resulting in a reduction in H3K9me3 and DNA methylation. Further investigation showed that decreased DNA methylation of differentiation genes, such as *Gata3*, *Peg3*, *Socs3*, and *Hand1*, was the main cause of their increased expression during differentiation (126). Furthermore, histone lactylation has also been found to play a regulatory role in trophoblast cells. In patient with preeclampsia, the placenta showed a significant increase in the H3K18la modification compared to the control group. The activation of target genes *FN1* and *SERPINE1* by H3K18la promotes placental fibrosis (127), indicating the functional significance of histone lactylation in placental pathology.

## Concluding remarks

In recent years, emerging evidence has underscored the significance of epigenetic regulations during embryo implantation and placentation, highlighting their crucial role in these processes. Here, we focused specifically on histone modifications and summarized the key findings in this field. Despite increased knowledge on the topic, there are still many unknowns regarding the molecular basis of histone modifications and their roles in the interaction between the embryo and the uterus during gestation. Specifically, while considerable attention has been given to histone methylation and acetylation in the context of embryo implantation and placentation, further investigations are required to fully unravel the complexities of the epigenetic regulatory network involved, encompassing other types of modifications as well. In addition, despite the increasing studies of various histone modification factors at the maternal-fetal interface, comprehending the physiological and pathological functions, as well as the underlying mechanisms of these factors, remain major challenges in this field.

Moreover, the existing models used in relevant research have certain limitations, primarily relying on traditional cell lines and conditional knockout mouse models. Unfortunately, many essential genes crucial for implantation and placentation cannot be thoroughly studied, as their knockout often leads to embryonic lethality or developmental defects. Hence, there is an urgent need to develop inducible Cre systems with uterus/placenta-specific gene promoters, enabling a more precise assessment of histone modifications throughout different stages of pregnancy. Additionally, considering the disparities between mice and humans, it is imperative to develop more diverse models, such as organoids and cell chips, to overcome the current limitations and effectively identify therapeutic targets for diseases such as implantation failure, preeclampsia, and fetal growth restriction.

## Author contributions

SB wrote the manuscript, ZT summarized the mouse models and prepared the figure, SZ and DC discussed the outline and revised the manuscript. All authors contributed to the article and approved the submitted version.

## References

[1] Wang H Dey SK. Roadmap to embryo implantation: clues from mouse models. Nat Rev Genet (2006) 7:185–99. doi: 10.1038/nrg1808 16485018

[2] Zhang S Lin H Kong S Wang S Wang H Wang H. Physiological and molecular determinants of embryo implantation. Mol Aspects Med (2013) 34:939–80. doi: 10.1016/j.mam.2012.12.011 PMC4278353 23290997

[3] Hemberger M Hanna CW Dean W. Mechanisms of early placental development in mouse and humans. Nat Rev Genet (2020) 21:27–43. doi: 10.1038/s41576-019-0169-4 31534202

[4] Maltepe E Fisher SJ. Placenta: the forgotten organ. Annu Rev Cell Dev Biol (2015) 31:523–52. doi: 10.1146/annurev-cellbio-100814-125620 26443191

[5] Brosens I Pijnenborg R Vercruysse L Romero R. The "Great Obstetrical Syndromes" are associated with disorders of deep placentation. Am J Obstet Gynecol (2011) 204:193–201. doi: 10.1016/j.ajog.2010.08.009 21094932 PMC3369813

[6] Tsankova N Renthal W Kumar A Nestler EJ. Epigenetic regulation in psychiatric disorders. Nat Rev Neurosci (2007) 8:355–67. doi: 10.1038/nrn2132 17453016

[7] Pal S Tyler JK. Epigenetics and aging. Sci Adv (2016) 2. doi: 10.1126/sciadv.1600584 PMC4966880 27482540

[8] Millán-Zambrano G Burton A Bannister AJ Schneider R. Histone post-translational modifications - cause and consequence of genome function. Nat Rev Genet (2022) 23:563–80. doi: 10.1038/s41576-022-00468-7 35338361

[9] Kouzarides T. Histone-modifying enzymes. Cell (2007) 128:1227–36. doi: 10.1016/j.cell.2007.02.018 17320515

[10] Lewis PW Müller MM Koletsky MS Cordero F Lin S Banaszynski LA. Inhibition of PRC2 activity by a gain-of-function H3 mutation found in pediatric glioblastoma. Science (2013) 340:857–61. doi: 10.1126/science.1232245 PMC3951439 23539183

[11] Ronan JL Wu W Crabtree GR. From neural development to cognition: unexpected roles for chromatin. Nat Rev Genet (2013) 14:347–59. doi: 10.1038/nrg3413 PMC4010428 23568486

[12] Kong S Zhou C Bao H Ni Z Liu M He B. Epigenetic control of embryo-uterine crosstalk at peri-implantation. Cell Mol Life Sci (2019) 76:4813–28. doi: 10.1007/s00018-019-03245-8 PMC11105790 31352535

[13] Kim H Kim M Im S-K Fang S. Mouse Cre-LoxP system: general principles to determine tissue-specific roles of target genes. Lab Anim Res (2018) 34:147–59. doi: 10.5625/lar.2018.34.4.147 PMC6333611 30671100

[14] Soyal SM Mukherjee A Lee KYS Li J Li H Demayo FJ. Cre-mediated recombination in cell lineages that express the progesterone receptor. Genesis (2005) 41:58–66. doi: 10.1002/gene.20098 15682389

[15] Daikoku T Ogawa Y Terakawa J Ogawa A Defalco T Dey SK. Lactoferrin-iCre: a new mouse line to study uterine epithelial gene function. Endocrinology (2014) 155:2718–24. doi: 10.1210/en.2014-1265 PMC4060188 24823394

[16] Contreras CM Akbay EA Gallardo TD Haynie JM Sharma S Tagao O. Lkb1 inactivation is sufficient to drive endometrial cancers that are aggressive yet highly responsive to mTOR inhibitor monotherapy. Dis Model Mech (2010) 3:181–93. doi: 10.1242/dmm.004440 PMC2869492 20142330

[17] Winuthayanon W Hewitt SC Orvis GD Behringer RR Korach KS. Uterine epithelial estrogen receptor α is dispensable for proliferation but essential for complete biological and biochemical responses. Proc Natl Acad Sci USA (2010) 107:19272–7. doi: 10.1073/pnas.1013226107 PMC2984169 20974921

[18] Jorgez CJ Klysik M Jamin SP Behringer RR Matzuk MM. Granulosa cell-specific inactivation of follistatin causes female fertility defects. Mol Endocrinol (2004) 18:953–67. doi: 10.1210/me.2003-0301 14701941

[19] Regan CP Manabe I Owens GK. Development of a smooth muscle–targeted cre recombinase mouse reveals novel insights regarding smooth muscle myosin heavy chain promoter regulation. Circ Res (2000) 87:363–9. doi: 10.1161/01.RES.87.5.363 10969033

[20] Holtwick R Gotthardt M Skryabin B Steinmetz M Potthast R Zetsche B. Smooth muscle-selective deletion of guanylyl cyclase-A prevents the acute but not chronic effects of ANP on blood pressure. Proc Natl Acad Sci USA (2002) 99:7142–7. doi: 10.1073/pnas.102650499 PMC124542 11997476

[21] Margueron R Reinberg D. The Polycomb complex PRC2 and its mark in life. Nature (2011) 469:343–9. doi: 10.1038/nature09784 PMC3760771 21248841

[22] Katoh N Kuroda K Tomikawa J Ogata-Kawata H Ozaki R Ochiai A. Reciprocal changes of H3K27ac and H3K27me3 at the promoter regions of the critical genes for endometrial decidualization. Epigenomics (2018) 10:1243–57. doi: 10.2217/epi-2018-0006 30212243

[23] Grimaldi G Christian M Steel JH Henriet P Poutanen M Brosens JJ. Down-regulation of the histone methyltransferase EZH2 contributes to the epigenetic programming of decidualizing human endometrial stromal cells. Mol Endocrinol (2011) 25:1892–903. doi: 10.1210/me.2011-1139 PMC3198959 21903722

[24] Nancy P Siewiera J Rizzuto G Tagliani E Osokine I Manandhar P. H3K27me3 dynamics dictate evolving uterine states in pregnancy and parturition. J Clin Invest (2018) 128:233–47. doi: 10.1172/JCI95937 PMC5749543 29202469

[25] Kim KH Roberts CW. Targeting EZH2 in cancer. Nat Med (2016) 22:128–34. doi: 10.1038/nm.4036 PMC4918227 26845405

[26] Fang X Ni N Lydon JP Ivanov I Bayless KJ Rijnkels M. Enhancer of Zeste 2 polycomb repressive complex 2 subunit is required for uterine epithelial integrity. Am J Pathol (2019) 189:1212–25. doi: 10.1016/j.ajpath.2019.02.016 PMC6547058 30954472

[27] Nanjappa MK Mesa AM Medrano TI Jefferson WN Demayo FJ Williams CJ. The histone methyltransferase EZH2 is required for normal uterine development and function in mice. Biol Reprod (2019) 101:306–17. doi: 10.1093/biolre/ioz097 PMC7302517 31201420

[28] Osokine I Siewiera J Rideaux D Ma S Tsukui T Erlebacher A. Gene silencing by EZH2 suppresses TGF-β activity within the decidua to avert pregnancy-adverse wound healing at the maternal-fetal interface. Cell Rep (2022) 38:110329. doi: 10.1016/j.celrep.2022.110329 35108527 PMC8833843

[29] Fukui Y Hirota Y Aikawa S Sakashita A Shimizu-Hirota R Takeda N. The EZH2-PRC2-H3K27me3 axis governs the endometrial cell cycle and differentiation for blastocyst invasion. Cell Death Dis (2023) 14:320. doi: 10.1038/s41419-023-05832-x 37198149 PMC10192223

[30] Ni N Jalufka FL Fang X Mccreedy DA Li Q. Role of EZH2 in uterine gland development. Int J Mol Sci (2022) 23:15665. doi: 10.3390/ijms232415665 36555314 PMC9779349

[31] Santos-Rosa H Schneider R Bannister AJ Sherriff J Bernstein BE Emre NT. Active genes are tri-methylated at K4 of histone H3. Nature (2002) 419:407–11. doi: 10.1038/nature01080 12353038

[32] Dreijerink KM Groner AC Vos ES Font-Tello A Gu L Chi D. Enhancer-mediated oncogenic function of the menin tumor suppressor in breast cancer. Cell Rep (2017) 18:2359–72. doi: 10.1016/j.celrep.2017.02.025 PMC5609449 28273452

[33] Liu M Deng W Tang L Liu M Bao H Guo C. Menin directs regionalized decidual transformation through epigenetically setting PTX3 to balance FGF and BMP signaling. Nat Commun (2022) 13:1006. doi: 10.1038/s41467-022-28657-2 35194044 PMC8864016

[34] Cui T He B Kong S Zhou C Zhang H Ni Z. PR-Set7 deficiency limits uterine epithelial population growth hampering postnatal gland formation in mice. Cell Death Differ (2017) 24:2013–21. doi: 10.1038/cdd.2017.120 PMC5686342 28731465

[35] Libby PR. Histone acetylation and hormone action. Early effects of oestradiol-17β on histone acetylation in rat uterus. Biochem J (1972) 130:663–9. doi: 10.1042/bj1300663 PMC1174504 4664926

[36] Serra MJ Ledford BE Baggett B. Synthesis and modification of the histones during the decidual cell reaction in the mouse uterus. Biol Reprod (1979) 20:214–20. doi: 10.1095/biolreprod20.2.214 454732

[37] Gunin AG Kapitova IN Suslonova NV. Effects of histone deacetylase inhibitors on estradiol-induced proliferation and hyperplasia formation in the mouse uterus. J Endocrinol (2005) 185:539–50. doi: 10.1677/joe.1.06118 15930180

[38] Uchida H Maruyama T Nagashima T Asada H Yoshimura Y. Histone deacetylase inhibitors induce differentiation of human endometrial adenocarcinoma cells through up-regulation of glycodelin. Endocrinology (2005) 146:5365–73. doi: 10.1210/en.2005-0359 16123169

[39] Tamura I Jozaki K Sato S Shirafuta Y Shinagawa M Maekawa R. The distal upstream region of insulin-like growth factor–binding protein-1 enhances its expression in endometrial stromal cells during decidualization. J Biol Chem (2018) 293:5270–80. doi: 10.1074/jbc.RA117.000234 PMC5892597 29453285

[40] Amin SA Adhikari N Jha T Ghosh B. Designing potential HDAC3 inhibitors to improve memory and learning. J Biomol Struct Dyn (2019) 37:2133–42. doi: 10.1080/07391102.2018.1477625 30044204

[41] Kim TH Yoo J-Y Choi K-C Shin J-H Leach RE Fazleabas AT. Loss of HDAC3 results in nonreceptive endometrium and female infertility. Sci Transl Med (2019) 11. doi: 10.1126/scitranslmed.aaf7533 PMC6650287 30626716

[42] Estella C Herrer I Atkinson SP Quiñonero A Martínez S Pellicer A. Inhibition of histone deacetylase activity in human endometrial stromal cells promotes extracellular matrix remodelling and limits embryo invasion. PLoS One (2012) 7. doi: 10.1371/journal.pone.0030508 PMC3266920 22291969

[43] Xu C Wang L Fozouni P Evjen G Chandra V Jiang J. SIRT1 is downregulated by autophagy in senescence and ageing. Nat Cell Biol (2020) 22:1170–9. doi: 10.1038/s41556-020-00579-5 PMC7805578 32989246

[44] Sauve AA Wolberger C Schramm VL Boeke JD. The biochemistry of sirtuins. Annu Rev Biochem (2006) 75:435–65. doi: 10.1146/annurev.biochem.74.082803.133500 16756498

[45] Vaquero A Scher M Lee D Erdjument-Bromage H Tempst P Reinberg D. Human SirT1 interacts with histone H1 and promotes formation of facultative heterochromatin. Mol Cell (2004) 16:93–105. doi: 10.1016/j.molcel.2004.08.031 15469825

[46] Nogueiras R Habegger KM Chaudhary N Finan B Banks AS Dietrich MO. Sirtuin 1 and sirtuin 3: physiological modulators of metabolism. Physiol Rev (2012) 92(3):1479–514. doi: 10.1152/physrev.00022.2011 PMC3746174 22811431

[47] Cheng H-L Mostoslavsky R Saito SI Manis JP Gu Y Patel P. Developmental defects and p53 hyperacetylation in Sir2 homolog (SIRT1)-deficient mice. Proc Natl Acad Sci USA (2003) 100:10794–9. doi: 10.1073/pnas.1934713100 PMC196882 12960381

[48] Bouras T Fu M Sauve AA Wang F Quong AA Perkins ND. SIRT1 deacetylation and repression of p300 involves lysine residues 1020/1024 within the cell cycle regulatory domain 1. J Biol Chem (2005) 280:10264–76. doi: 10.1074/jbc.M408748200 15632193

[49] Wang J Chen J. SIRT1 regulates autoacetylation and histone acetyltransferase activity of TIP60. J Biol Chem (2010) 285:11458–64. doi: 10.1074/jbc.M109.087585 PMC2857024 20100829

[50] Fujita Y Yamashita T. Sirtuins in neuroendocrine regulation and neurological diseases. Front Neurosci (2018) 12:778. doi: 10.3389/fnins.2018.00778 30416425 PMC6213750

[51] Yu Q Dong L Li Y Liu G. SIRT1 and HIF1α signaling in metabolism and immune responses. Cancer Lett (2018) 418:20–6. doi: 10.1016/j.canlet.2017.12.035 29306019

[52] Cummings MJ Yu H Paudel S Hu G Li X Hemberger M. Uterine-specific SIRT1 deficiency confers premature uterine aging and impairs invasion and spacing of blastocyst, and stromal cell decidualization, in mice. Mol Hum Reprod (2022) 28:gaac016. doi: 10.1093/molehr/gaac016 35536234 PMC10689003

[53] Hwang YJ Sung G-J Marquardt R Young SL Lessey BA Kim TH. SIRT1 plays an important role in implantation and decidualization during mouse early pregnancy. Biol Reprod (2022) 106:1072–82. doi: 10.1093/biolre/ioac026 PMC9198957 35134122

[54] Kim TH Young SL Sasaki T Deaton JL Schammel DP Palomino WA. Role of SIRT1 and progesterone resistance in normal and abnormal endometrium. J Clin Endocrinol Metab (2022) 107:788–800. doi: 10.1210/clinem/dgab753 34665857 PMC8851922

[55] Süssmuth SD Haider S Landwehrmeyer GB Farmer R Frost C Tripepi G. An exploratory double-blind, randomized clinical trial with selisistat, a SirT1 inhibitor, in patients with H untington's disease. Br J Clin Pharmacol (2015) 79:465–76. doi: 10.1111/bcp.12512 PMC4345957 25223731

[56] Tamura I Asada H Maekawa R Tanabe M Lee L Taketani T. Induction of IGFBP-1 expression by cAMP is associated with histone acetylation status of the promoter region in human endometrial stromal cells. Endocrinology (2012) 153:5612–21. doi: 10.1210/en.2012-1420 23011923

[57] Tamura I Ohkawa Y Sato T Suyama M Jozaki K Okada M. Genome-wide analysis of histone modifications in human endometrial stromal cells. Mol Endocrinol (2014) 28:1656–69. doi: 10.1210/me.2014-1117 PMC5414786 25073104

[58] Uchida H Maruyama T Ohta K Ono M Arase T Kagami M. Histone deacetylase inhibitor-induced glycodelin enhances the initial step of implantation. Hum Reprod (2007) 22:2615–22. doi: 10.1093/humrep/dem263 17720699

[59] Vitiello D Kodaman PH Taylor HS. HOX genes in implantation. Semin Reprod Med (2007) 25:431–6. doi: 10.1055/s-2007-991040 17960527

[60] Zhu L-H Sun L-H Hu Y-L Jiang Y Liu H-Y Shen X-Y. PCAF impairs endometrial receptivity and embryo implantation by down-regulating β3-integrin expression *via* HOXA10 acetylation. J Clin Endocrinol Metab (2013) 98:4417–28. doi: 10.1210/jc.2013-1429 24037888

[61] De Napoles M Mermoud JE Wakao R Tang YA Endoh M Appanah R. Polycomb group proteins Ring1A/B link ubiquitylation of histone H2A to heritable gene silencing and X inactivation. Dev Cell (2004) 7:663–76. doi: 10.1016/j.devcel.2004.10.005 15525528

[62] Bian F Gao F Kartashov AV Jegga AG Barski A Das SK. Polycomb repressive complex 1 controls uterine decidualization. Sci Rep (2016) 6:26061. doi: 10.1038/srep26061 27181215 PMC4867636

[63] Xin Q Kong S Yan J Qiu J He B Zhou C. Polycomb subunit BMI1 determines uterine progesterone responsiveness essential for normal embryo implantation. J Clin Invest (2018) 128:175–89. doi: 10.1172/JCI92862 PMC5749512 29202468

[64] Zhang D Tang Z Huang H Zhou G Cui C Weng Y. Metabolic regulation of gene expression by histone lactylation. Nature (2019) 574:575–80. doi: 10.1038/s41586-019-1678-1 PMC6818755 31645732

[65] Yu J Chai P Xie M Ge S Ruan J Fan X. Histone lactylation drives oncogenesis by facilitating m(6)A reader protein YTHDF2 expression in ocular melanoma. Genome Biol (2021) 22:85. doi: 10.1186/s13059-021-02308-z 33726814 PMC7962360

[66] Xiong J He J Zhu J Pan J Liao W Ye H. Lactylation-driven METTL3-mediated RNA m(6)A modification promotes immunosuppression of tumor-infiltrating myeloid cells. Mol Cell (2022) 82:1660–1677.e10. doi: 10.1016/j.molcel.2022.02.033 35320754

[67] Yang Q Liu J Wang Y Zhao W Wang W Cui J. A proteomic atlas of ligand-receptor interactions at the ovine maternal-fetal interface reveals the role of histone lactylation in uterine remodeling. J Biol Chem (2022) 298:101456. doi: 10.1016/j.jbc.2021.101456 34861240 PMC8733267

[68] Cockburn K Rossant J. Making the blastocyst: lessons from the mouse. J Clin Invest (2010) 120:995–1003. doi: 10.1172/JCI41229 20364097 PMC2846056

[69] Paul S Knott JG. Epigenetic control of cell fate in mouse blastocysts: the role of covalent histone modifications and chromatin remodeling. Mol Reprod Dev (2014) 81:171–82. doi: 10.1002/mrd.22219 PMC4276566 23893501

[70] Yeap L-S Hayashi K Surani MA. ERG-associated protein with SET domain (ESET)-Oct4 interaction regulates pluripotency and represses the trophectoderm lineage. Epigenetics Chromatin (2009) 2:1–17. doi: 10.1186/1756-8935-2-12 19811652 PMC2763847

[71] Yuan P Han J Guo G Orlov YL Huss M Loh Y-H. Eset partners with Oct4 to restrict extraembryonic trophoblast lineage potential in embryonic stem cells. Genes Dev (2009) 23:2507–20. doi: 10.1101/gad.1831909 PMC2779752 19884257

[72] Alder O Lavial F Helness A Brookes E Pinho S Chandrashekran A. Ring1B and Suv39h1 delineate distinct chromatin states at bivalent genes during early mouse lineage commitment. Development (2010) 137:2483–92. doi: 10.1242/dev.048363 PMC2927698 20573702

[73] Rugg-Gunn PJ Cox BJ Ralston A Rossant J. Distinct histone modifications in stem cell lines and tissue lineages from the early mouse embryo. Proc Natl Acad Sci USA (2010) 107:10783–90. doi: 10.1073/pnas.0914507107 PMC2890770 20479220

[74] Kidder BL Palmer S. HDAC1 regulates pluripotency and lineage specific transcriptional networks in embryonic and trophoblast stem cells. Nucleic Acids Res (2012) 40:2925–39. doi: 10.1093/nar/gkr1151 PMC3326306 22156375

[75] Roberts RM Green JA Schulz LC. The evolution of the placenta. Reproduction (2016) 152:R179–89. doi: 10.1530/REP-16-0325 PMC5033709 27486265

[76] Turco MY Moffett A. Development of the human placenta. Development (2019) 146:dev163428. doi: 10.1242/dev.163428 31776138

[77] Knöfler M HAIDER S SALEH L POLLHEIMER J GAMAGE T JAMES J. Human placenta and trophoblast development: key molecular mechanisms and model systems. Cell Mol Life Sci (2019) 76:3479–96. doi: 10.1007/s00018-019-03104-6 PMC6697717 31049600

[78] Gude NM Roberts CT Kalionis B King RG. Growth and function of the normal human placenta. Thromb Res (2004) 114:397–407. doi: 10.1016/j.thromres.2004.06.038 15507270

[79] Watson ED Cross JC. Development of structures and transport functions in the mouse placenta. Physiol (Bethesda) (2005) 20:180–93. doi: 10.1152/physiol.00001.2005 15888575

[80] Kong S Liang G Tu Z Chen D Wang H Lu J. Generation of Elf5-Cre knockin mouse strain for trophoblast-specific gene manipulation. Genesis (2018) 56. doi: 10.1002/dvg.23101 29532590

[81] Calzonetti T Stevenson L Rossant J. A novel regulatory region is required for trophoblast-specific transcription in transgenic mice. Dev Biol (1995) 171:615–26. doi: 10.1006/dbio.1995.1309 7556941

[82] Nadeau V Guillemette S Bélanger L-F Jacob O Roy S Charron J. Map2k1 and Map2k2 genes contribute to the normal development of syncytiotrophoblasts during placentation. Development (2009) 136(8):1363–74. doi: 10.1242/dev.031872 19304888

[83] Wenzel PL Leone G. Expression of Cre recombinase in early diploid trophoblast cells of the mouse placenta. Genesis (2007) 45:129–34. doi: 10.1002/dvg.20276 17299749

[84] Zhou CC Chang J Mi T Abbasi S Gu D Huang L. Targeted expression of Cre recombinase provokes placental-specific DNA recombination in transgenic mice. PLoS One (2012) 7. doi: 10.1371/journal.pone.0029236 PMC3281813 22363401

[85] Ouseph MM Li J Chen H-Z Pécot T Wenzel P Thompson JC. Atypical E2F repressors and activators coordinate placental development. Dev Cell (2012) 22:849–62. doi: 10.1016/j.devcel.2012.01.013 PMC3483796 22516201

[86] Ozguldez HO Fan R Bedzhov I. Placental gene editing *via* trophectoderm-specific Tat-Cre/loxP recombination. Development (2020) 147:dev190371. doi: 10.1242/dev.190371 32541013

[87] Zheng H Huang B Zhang B Xiang Y Du Z Xu Q. Resetting epigenetic memory by reprogramming of histone modifications in mammals. Mol Cell (2016) 63:1066–79. doi: 10.1016/j.molcel.2016.08.032 27635762

[88] Inoue A Jiang L Lu F Suzuki T Zhang Y. Maternal H3K27me3 controls DNA methylation-independent imprinting. Nature (2017) 547:419–24. doi: 10.1038/nature23262 PMC9674007 28723896

[89] Yang X Hu B Hou Y Qiao Y Wang R Chen Y. Silencing of developmental genes by H3K27me3 and DNA methylation reflects the discrepant plasticity of embryonic and extraembryonic lineages. Cell Res (2018) 28:593–6. doi: 10.1038/s41422-018-0010-1 PMC5951795 29463899

[90] Yu Y Li X Jiao R Lu Y Jiang X Li X. H3K27me3-H3K4me1 transition at bivalent promoters instructs lineage specification in development. Cell Biosci (2023) 13:66. doi: 10.1186/s13578-023-01017-3 36991495 PMC10061859

[91] Lewis A Mitsuya K Umlauf D Smith P Dean W Walter J. Imprinting on distal chromosome 7 in the placenta involves repressive histone methylation independent of DNA methylation. Nat Genet (2004) 36:1291–5. doi: 10.1038/ng1468 15516931

[92] Umlauf D Goto Y Cao R Cerqueira F Wagschal A Zhang Y. Imprinting along the Kcnq1 domain on mouse chromosome 7 involves repressive histone methylation and recruitment of Polycomb group complexes. Nat Genet (2004) 36:1296–300. doi: 10.1038/ng1467 15516932

[93] Wagschal A Sutherland HG Woodfine K Henckel A Chebli K Schulz R. G9a histone methyltransferase contributes to imprinting in the mouse placenta. Mol Cell Biol (2008) 28:1104–13. doi: 10.1128/MCB.01111-07 PMC2223396 18039842

[94] Chen Z Yin Q Inoue A Zhang C Zhang Y. Allelic H3K27me3 to allelic DNA methylation switch maintains noncanonical imprinting in extraembryonic cells. Sci Adv (2019) 5. doi: 10.1126/sciadv.aay7246 PMC6989337 32064321

[95] Wang J Mager J Schnedier E Magnuson T. The mouse PcG gene eed is required for Hox gene repression and extraembryonic development. Mamm Genome (2002) 13:493–503. doi: 10.1007/s00335-002-2182-7 12370779

[96] Matoba S Wang H Jiang L Lu F Iwabuchi KA Wu X. Loss of H3K27me3 imprinting in somatic cell nuclear transfer embryos disrupts post-implantation development. Cell Stem Cell (2018) 23:343–354. e5. doi: 10.1016/j.stem.2018.06.008 30033120 PMC6326833

[97] Inoue K Ogonuki N Kamimura S Inoue H Matoba S Hirose M. Loss of H3K27me3 imprinting in the Sfmbt2 miRNA cluster causes enlargement of cloned mouse placentas. Nat Commun (2020) 11:2150. doi: 10.1038/s41467-020-16044-8 32358519 PMC7195362

[98] Lv S Wang N Lv H Yang J Liu J Li W-P. The attenuation of trophoblast invasion caused by the downregulation of EZH2 is involved in the pathogenesis of human recurrent miscarriage. Mol Ther Nucleic Acids (2019) 14:377–87. doi: 10.1016/j.omtn.2018.12.011 PMC6356049 30710891

[99] Nugent BM O’donnell CM Epperson CN Bale TL. Placental H3K27me3 establishes female resilience to prenatal insults. Nat Commun (2018) 9:2555. doi: 10.1038/s41467-018-04992-1 29967448 PMC6028627

[100] Chakraborty D Cui W Rosario GX Scott RL Dhakal P Renaud SJ. HIF-KDM3A-MMP12 regulatory circuit ensures trophoblast plasticity and placental adaptations to hypoxia. Proc Natl Acad Sci USA (2016) 113:E7212–21. doi: 10.1073/pnas.1612626113 PMC5135375 27807143

[101] Wang L Chakraborty D Iqbal K Soares MJ. SUV39H2 controls trophoblast stem cell fate. Biochim Biophys Acta Gen Subj (2021) 1865:129867. doi: 10.1016/j.bbagen.2021.129867 33556426 PMC8052280

[102] Wang H Fan Z Shliaha PV Miele M Hendrickson RC Jiang X. H3K4me3 regulates RNA polymerase II promoter-proximal pause-release. Nature (2023) 615:339–48. doi: 10.1038/s41586-023-05780-8 PMC9995272 36859550

[103] Xu J Kidder BL. KDM5B decommissions the H3K4 methylation landscape of self-renewal genes during trophoblast stem cell differentiation. Biol Open (2018) 7:bio031245. doi: 10.1242/bio.031245 29748167 PMC5992522

[104] Shi Y Lan F Matson C Mulligan P Whetstine JR Cole PA. Histone demethylation mediated by the nuclear amine oxidase homolog LSD1. Cell (2004) 119:941–53. doi: 10.1016/j.cell.2004.12.012 15620353

[105] Metzger E Wissmann M Yin N Müller JM Schneider R Peters AH. LSD1 demethylates repressive histone marks to promote androgen-receptor-dependent transcription. Nature (2005) 437:436–9. doi: 10.1038/nature04020 16079795

[106] Zhu D Hölz S Metzger E Pavlovic M Jandausch A Jilg C. Lysine-specific demethylase 1 regulates differentiation onset and migration of trophoblast stem cells. Nat Commun (2014) 5:3174. doi: 10.1038/ncomms4174 24448552

[107] Castex J Willmann D Kanouni T Arrigoni L Li Y Friedrich M. Inactivation of Lsd1 triggers senescence in trophoblast stem cells by induction of Sirt4. Cell Death Dis (2017) 8. doi: 10.1038/cddis.2017.48 PMC5386490 28230862

[108] Mak KH-M Lam YM Ng RK. Histone demethylase JMJD2B/KDM4B regulates transcriptional program *via* distinctive epigenetic targets and protein interactors for the maintenance of trophoblast stem cells. Sci Rep (2021) 11:884. doi: 10.1038/s41598-020-79601-7 33441614 PMC7806742

[109] Bai D Sun J Chen C Jia Y Li Y Liu K. Aberrant H3K4me3 modification of epiblast genes of extraembryonic tissue causes placental defects and implantation failure in mouse IVF embryos. Cell Rep (2022) 39:110784. doi: 10.1016/j.celrep.2022.110784 35508139

[110] Meister S Hahn L Beyer S Kuhn C Jegen M Von Schonfeldt V. Epigenetic modification *via* H3K4me3 and H3K9ac in human placenta is reduced in preeclampsia. J Reprod Immunol (2021) 145:103287. doi: 10.1016/j.jri.2021.103287 33662848

[111] Io S Kabata M Iemura Y Semi K Morone N Minagawa A. Capturing human trophoblast development with naive pluripotent stem cells *in vitro* . Cell Stem Cell (2021) 28:1023–1039. e13. doi: 10.1016/j.stem.2021.03.013 33831365

[112] Yu S Shen Q Zhang R Wu X Zhang J Zhao W. KDM4C contributes to trophoblast-like stem cell conversion from porcine-induced pluripotent stem cells (piPSCs) *via* regulating CDX2. Int J Mol Sci (2022) 23:7586. doi: 10.3390/ijms23147586 35886932 PMC9323581

[113] Okae H Toh H Sato T Hiura H Takahashi S Shirane K. Derivation of human trophoblast stem cells. Cell Stem Cell (2018) 22:50–63. e6. doi: 10.1016/j.stem.2017.11.004 29249463

[114] Jaju Bhattad G Jeyarajah MJ Mcgill MG Dumeaux V Okae H Arima T. Histone deacetylase 1 and 2 drive differentiation and fusion of progenitor cells in human placental trophoblasts. Cell Death Dis (2020) 11:311. doi: 10.1038/s41419-020-2500-6 32366868 PMC7198514

[115] Hepp P Hutter S Knabl J Hofmann S Kuhn C Mahner S. Histone H3 lysine 9 acetylation is downregulated in GDM Placentas and Calcitriol supplementation enhanced this effect. Int J Mol Sci (2018) 19:4061. doi: 10.3390/ijms19124061 30558244 PMC6321349

[116] Paauw N Lely A Joles J Franx A Nikkels P Mokry M. H3K27 acetylation and gene expression analysis reveals differences in placental chromatin activity in fetal growth restriction. Clin Epigenetics (2018) 10:1–11. doi: 10.1186/s13148-018-0508-x 29983832 PMC6020235

[117] Zhang B Kim MY Elliot G Zhou Y Zhao G Li D. Human placental cytotrophoblast epigenome dynamics over gestation and alterations in placental disease. Dev Cell (2021) 56:1238–1252. e5. doi: 10.1016/j.devcel.2021.04.001 33891899 PMC8650129

[118] Wang Y Zhang Y Wu Y He Y Xiang J Huang J. SIRT1 regulates trophoblast senescence in premature placental aging in preeclampsia. Placenta (2022) 122:56–65. doi: 10.1016/j.placenta.2022.04.001 35460951

[119] Rajan KAN Khater M Soncin F Pizzo D Moretto-Zita M Pham J. Sirtuin1 is required for proper trophoblast differentiation and placental development in mice. Placenta (2018) 62:1–8. doi: 10.1016/j.placenta.2017.12.002 29405961 PMC5805473

[120] Xiong L Ye X Chen Z Fu H Li S Xu P. Advanced maternal age-associated SIRT1 deficiency compromises trophoblast epithelial– mesenchymal transition through an increase in vimentin acetylation. Aging Cell (2021) 20. doi: 10.1111/acel.13491 PMC8520724 34605151

[121] Mobley RJ Raghu D Duke LD Abell-Hart K Zawistowski JS Lutz K. MAP3K4 controls the chromatin modifier HDAC6 during trophoblast stem cell epithelial-to-mesenchymal transition. Cell Rep (2017) 18:2387–400. doi: 10.1016/j.celrep.2017.02.030 PMC5496714 28273454

[122] Dancy BM Cole PA. Protein lysine acetylation by p300/CBP. Chem Rev (2015) 115:2419–52. doi: 10.1021/cr500452k PMC4378506 25594381

[123] Abell AN Jordan NV Huang W Prat A Midland AA Johnson NL. MAP3K4/CBP-regulated H2B acetylation controls epithelial-mesenchymal transition in trophoblast stem cells. Cell Stem Cell (2011) 8:525–37. doi: 10.1016/j.stem.2011.03.008 PMC3091002 21549327

[124] Li J Wang W Liu C Wang W Li W Shu Q. Critical role of histone acetylation by p300 in human placental 11beta-HSD2 expression. J Clin Endocrinol Metab (2013) 98:E1189–97. doi: 10.1210/jc.2012-4291 23714681

[125] Wang S Wang Y. Peptidylarginine deiminases in citrullination, gene regulation, health and pathogenesis. Biochim Biophys Acta (2013) 1829:1126–35. doi: 10.1016/j.bbagrm.2013.07.003 PMC3775966 23860259

[126] Ballasy NN Bering EA Kokorudz C Radford BN Zhao X Dean W. Padi2/3 deficiency alters the epigenomic landscape and causes premature differentiation of mouse trophoblast stem cells. Cells (2022) 11:2466. doi: 10.3390/cells11162466 36010543 PMC9406452

[127] Li X Yang N Wu Y Wang X Sun J Liu L. Hypoxia regulates fibrosis-related genes *via* histone lactylation in the placentas of patients with preeclampsia. J Hypertens (2022) 40:1189–98. doi: 10.1097/HJH.0000000000003129 35703881

